# Evaluation of Antiviral Efficacy of Ribavirin, Arbidol, and T-705 (Favipiravir) in a Mouse Model for Crimean-Congo Hemorrhagic Fever

**DOI:** 10.1371/journal.pntd.0002804

**Published:** 2014-05-01

**Authors:** Lisa Oestereich, Toni Rieger, Melanie Neumann, Christian Bernreuther, Maria Lehmann, Susanne Krasemann, Stephanie Wurr, Petra Emmerich, Xavier de Lamballerie, Stephan Ölschläger, Stephan Günther

**Affiliations:** 1 Department of Virology, Bernhard-Nocht-Institute for Tropical Medicine, Hamburg, Germany; 2 German Centre for Infection Research (DZIF), Hamburg, Germany; 3 Mouse Pathology Core Facility, University Medical Center Hamburg-Eppendorf, Hamburg, Germany; 4 Institute of Neuropathology, University Medical Center Hamburg-Eppendorf, Hamburg, Germany; 5 Aix Marseille Université, IRD French Institute of Research for Development, EHESP French School of Public Health, UMR_D 190 “Emergence des Pathologies Virales”, Marseille, France; Centre for Cellular and Molecular Biology (CCMB), India

## Abstract

**Background:**

Mice lacking the type I interferon receptor (IFNAR^−/−^ mice) reproduce relevant aspects of Crimean-Congo hemorrhagic fever (CCHF) in humans, including liver damage. We aimed at characterizing the liver pathology in CCHF virus-infected IFNAR^−/−^ mice by immunohistochemistry and employed the model to evaluate the antiviral efficacy of ribavirin, arbidol, and T-705 against CCHF virus.

**Methodology/Principal Findings:**

CCHF virus-infected IFNAR^−/−^ mice died 2–6 days post infection with elevated aminotransferase levels and high virus titers in blood and organs. Main pathological alteration was acute hepatitis with extensive bridging necrosis, reactive hepatocyte proliferation, and mild to moderate inflammatory response with monocyte/macrophage activation. Virus-infected and apoptotic hepatocytes clustered in the necrotic areas. Ribavirin, arbidol, and T-705 suppressed virus replication *in vitro* by ≥3 log units (IC_50_ 0.6–2.8 µg/ml; IC_90_ 1.2–4.7 µg/ml). Ribavirin [100 mg/(kg×d)] did not increase the survival rate of IFNAR^−/−^ mice, but prolonged the time to death (p<0.001) and reduced the aminotransferase levels and the virus titers. Arbidol [150 mg/(kg×d)] had no efficacy *in vivo*. Animals treated with T-705 at 1 h [15, 30, and 300 mg/(kg×d)] or up to 2 days [300 mg/(kg×d)] post infection survived, showed no signs of disease, and had no virus in blood and organs. Co-administration of ribavirin and T-705 yielded beneficial rather than adverse effects.

**Conclusions/Significance:**

Activated hepatic macrophages and monocyte-derived cells may play a role in the proinflammatory cytokine response in CCHF. Clustering of infected hepatocytes in necrotic areas without marked inflammation suggests viral cytopathic effects. T-705 is highly potent against CCHF virus *in vitro* and *in vivo*. Its *in vivo* efficacy exceeds that of the current standard drug for treatment of CCHF, ribavirin.

## Author Summary

Crimean-Congo hemorrhagic fever (CCHF) is endemic in Africa, Asia, southeast Europe, and the Middle East. The case fatality rate is 30–50%. Studies on pathophysiology and treatment of CCHF have been hampered by the lack of an appropriate animal model. We have employed CCHF virus-infected transgenic mice, which are defective in the innate immune response, as a disease model. These mice die from the infection and show signs of disease similar to those found in humans. First, we studied the liver pathology in the animals, as hepatic necrosis is a prominent feature of human CCHF. Secondly, we used the model to test the efficacy of antiviral drugs that are in clinical use or in an advanced stage of clinical testing. Besides ribavirin, the standard drug for treatment of CCHF, we tested arbidol, a drug in clinical use against respiratory infections, and T-705, a new drug in clinical development for the treatment of influenza virus infection. While ribavirin and arbidol showed some or no beneficial effect, respectively, T-705 was highly efficacious in the animal model. These data hold promise for clinical efficacy of T-705 in human CCHF.

## Introduction

Crimean-Congo hemorrhagic fever virus (CCHFV) is a negative-strand RNA virus belonging to the genus *Nairovirus* of the family *Bunyaviridae*. The virus is endemic in Africa, Asia, southeast Europe, and the Middle East. *Hyalomma* ticks transmit the virus to humans, wildlife, and livestock. Humans may also be infected by contact with infected livestock. Human-to-human transmission occurs mainly in the hospital setting. In humans, the virus causes a febrile illness that may be associated with hemorrhage, liver necrosis, shock, and multiorgan failure. Further hallmarks of the disease are increased levels of serum aspartate and alanine aminotransferase (AST and ALT, respectively), thrombocytopenia, and disseminated intravascular coagulopathy. The average case fatality rate is 30–50%, but may be higher in nosocomial outbreaks [1]–[5].

The pathophysiology of the disease is poorly understood. Endothelial and liver cell damage, induction of proinflammatory cytokines, and dysregulation of the coagulation cascade are thought to play a role [3]–[8]. Studies on the pathophysiology of Crimean-Congo hemorrhagic fever (CCHF) have been hampered by the lack of an appropriate animal model, as no mammal with fully functional immune system has been described so far — except humans — that develops disease upon infection. The first animal model was neonatal mouse [9]. Recently, two transgenic mouse models for CCHF have been described, first, mice lacking the signal transducer and activator of transcription 1 (STAT1^−/−^ mice) and second, mice lacking the type I (alpha/beta) interferon receptor (IFNAR^−/−^ mice) [10]–[12]. Both knockout mice are defective in the innate immune response, die rapidly from CCHFV infection, and reproduce relevant aspects of human CCHF. Surrogate models for CCHF employ IFNAR^−/−^ mice infected with Dugbe or Hazara virus [13], [14], two CCHFV-related nairoviruses that are not known to cause disease in human. Work with these models can be carried out at biosafety level (BSL)-2, while work with infectious CCHFV requires BSL-4 facilities.

In the present study, we aimed at characterizing the pathological changes in the liver of CCHFV-infected IFNAR^−/−^ mice in more detail. Furthermore, we employed this model to evaluate the antiviral efficacy of ribavirin, arbidol, and T-705 (favipiravir) against CCHFV *in vivo*. These drugs are either in clinical use or in an advanced stage of clinical testing. Ribavirin inhibits CCHFV replication in cell culture [15] and is administered to CCHF patients, though its clinical benefit is not proven and discussed controversially [16]–[19]. It shows beneficial effects in the neonatal and STAT1^−/−^ mouse models [9], [10]. Ribavirin currently is the only drug available for treatment of CCHF. Arbidol is a broad-spectrum antiviral showing activity against a range of RNA viruses *in vitro* and *in vivo*, most notably influenza A virus [20]–[24]. In Russia and China, the drug is in clinical use primarily for prophylaxis and treatment of acute respiratory infections including influenza. Arbidol is assumed to act via hydrophobic interactions with membranes and virus proteins, thus inhibiting viral fusion and entry [25]–[27]. T-705 is a potent inhibitor *in vitro* and in animal models of influenza virus, phleboviruses, hantaviruses, arenaviruses, alphaviruses, picornaviruses, and norovirus [28]–[35]. Following conversion to T-705-ribofuranosyl-5′-triphosphate, it presumably acts as a nucleotide analog that selectively inhibits the viral RNA-dependent RNA polymerase or causes lethal mutagenesis upon incorporation into the virus RNA [36]–[40]. T-705 (favipiravir) is currently in late stage clinical development for the treatment of influenza virus infection.

## Materials and Methods

### Ethics statement

This study was carried out in strict accordance with the recommendations of the German Society for Laboratory Animal Science under supervision of a veterinarian. The protocol was approved by the Committee on the Ethics of Animal Experiments of the City of Hamburg (Permit no. 44/11). All efforts were made to minimize the number of animals used for the experiments and suffering of the animals during the experiments. All staff carrying out animal experiments has passed an education and training program according to category B or C of the Federation of European Laboratory Animal Science Associations. The animal experiments in this study are reported according to the ARRIVE guidelines [41]. A total of 162 mice were used for this study and all mice were included in the analysis.

### Viruses

CCHFV strain Afg-09 2990 had been isolated in 2009 in our laboratory from a patient with a fatal course of infection [42] and passaged 2 times before it has been used in this study. The virus stock was grown on Vero E6 cells, quantified by immunofocus assay (see below), and stored at −70°C until use in *in vitro* and *in vivo* experiments.

### Antiviral compounds

Ribavirin (CAS no. 36791-04-5; PubChem CID 37542) was obtained from MP Biomedicals (order no. 02196066), arbidol hydrochloride (CAS no. 131707-23-8; PubChem CID 131410) from Waterstone Technology, USA (order no. 49823), and T-705 (favipiravir; CAS no. 259793-96-9; PubChem CID 492405) was custom synthesized by BOC Sciences, Creative Dynamics, USA.

### Antiviral and toxicity testing in cell culture

The compounds were dissolved in dimethyl sulfoxide (DMSO) at a concentration of about 10 mg/ml and stored at −20°C. Final DMSO concentration in the cell culture supernatant was 0.1%. Vero E6 cells were grown in Dulbecco's Modified Eagle's Medium (DMEM) (PAA Laboratories) supplemented with 5% fetal calf serum (FCS) and streptomycin/penicillin and seeded at a density of 4×10^4^ cells per well of a 24-well plate at 1 day before infection. Cells were inoculated with CCHFV at a multiplicity of infection (MOI) of 0.01 in the BSL-4 laboratory. The inoculum was removed after 1 h and replaced by fresh medium complemented with different concentrations of compound. For arbidol experiments, cells were additionally pretreated with arbidol 18 h before infection. Concentration in cell culture supernatant of infectious virus particles was measured 2–4 days post infection (p.i.) by immunofocus assay. Cell growth and viability under compound treatment was determined by the 3-(4,5-dimethylthiazol-2-yl)-2,5-diphenyl-2H-tetrazoliumbromide (MTT) method as described [43]. A sigmoidal dose–response curve was fitted to the data using Prism GraphPad 6.0 (GraphPad Software). The inhibitory concentrations that reduced the virus titer by 50%, 90%, and 99% (IC_50_, IC_90_, and IC_99_, respectively) and the cytotoxic concentrations that reduced cell growth by 50% and 90% (CC_50_ and CC_90_, respectively) were calculated from the sigmoidal functions.

For analysis of combinations of two drugs, an 8×8 concentration matrix was tested. Drugs *x* and *y* were tested in the concentrations *c* = 0; IC_90_/8; IC_90_/4; IC_90_/2; IC_90_; IC_90_•2; IC_90_•4; IC_90_•8 in all possible combinations (*c_x_*,*c_y_*). The IC_90_ values were derived from the prior single-drug experiments. The drug combination data were analyzed using the Bliss independence drug interaction model [44]. This model is defined by the equation *E_xy_* = *E_x_*+*E_y_*−(*E_x_*•*E_y_*), where *E_xy_* is the additive effect of drugs *x* and *y* as predicted by their individual effects *E_x_* and *E_y_*. *E_x_* = (*V_obs_*(0,0)−*V_obs_*(*c_x_*,0))/*V_obs_*(0,0) and *E_y_* = (*V_obs_*(0,0)−*V_obs_*(0,*c_y_*))/*V_obs_*(0,0), where *V_obs_*(*c_x_*,*c_y_*) is the observed, i.e. experimentally determined virus titer for (*c_x_*,*c_y_*). In analogy to the MacSynergy II program [44], [45], which evaluates antivirus data according to the Bliss independence model, a three-dimensional approach was used to identify areas where observed effects are greater (synergy) or less (antagonism) than those predicted by *E_xy_*. To this end, the ratio between predicted virus titer *V_pred_*(*c_x_*,*c_y_*) = *V_obs_*(0,0)•(1−*E_xy_*) and observed virus titer *V_obs_*(*c_x_*,*c_y_*) was calculated for each drug combination (*c_x_*,*c_y_*). A ratio >1 indicates synergy (i.e. for (*c_x_*,*c_y_*) the virus titer predicted for additive effect is higher than the experimentally determined virus titer), a ratio <1 indicates antagonism (i.e. for (*c_x_*,*c_y_*) the virus titer for predicted additive effect is lower than the experimentally determined virus titer).

### Mouse experiments

IFNAR^−/−^ mice (129Sv background) [46] were bred in the Specific Pathogen Free animal facility of the Bernhard-Nocht-Institute. Six to twelve-week-old female animals (weight median 20 g, range 15–24 g) were used for all experiments, except of nine males that were used for determination of the lethal virus dose. A group size of 5 animals was expected to provide sufficiently accurate estimates of survival rate, viremia, and clinical chemistry parameters. It allows to detect an 80% difference in survival rate between control and treatment group with p (alpha) = 0.05 and power (1 – beta) = 0.8. Experimental groups were age-matched. Three to five animals of a group were kept together in a conventional cage without enrichments. They had *ad libitum* access to food and water. Infection experiments with CCHFV were performed in the animal facility of the BSL-4 laboratory with artificial light/dark cycles.

Three to ten animals per group (depending on whether organ collection was planned) were infected by intraperitoneal (i.p.) injection with 0.3 to 10^4^ focus forming units (FFU) of CCHFV in 100 or 200 µl DMEM containing 2% FCS. The mode of administration was chosen to facilitate comparability with previously described CCHF mouse models [10], [11]. After infection, mice were monitored daily for signs of disease, and body weight and rectal body temperature were measured using thermometer BIO-TK8851 with BIO-BRET-3 rectal probe for mice (Bioseb, France). Animals with severe signs of disease such as seizures, bleeding, abdominal distention, diarrhea, agony, or weight loss of >15% within 2 days were euthanized. Blood samples of 30–80 µl per animal were drawn by tail vein puncture in intervals of 1–4 days over a period of 14 days (≤5 blood drawings in total) for clinical chemistry and viremia measurement. For organ collection, when criteria for euthanasia were fulfilled, and at the end of the experiment, animals were euthanized with an isoflurane overdose followed by cervical dislocation. Organs were collected after death or at day 3 p.i. from 2–3 animals that have been randomly chosen from experimental groups with 7–10 animals, and analyzed for infectious virus titer and histopathological changes. Experiments were not replicated.

### Antiviral and toxicity testing *in vivo*

Ribavirin was administered once daily by the i.p. route. A stock of 10 mg/ml in 0.9% NaCl was prepared before each application. Animals received a ribavirin dose of 100 mg/(kg×d) (200 µl for a 20-g mouse) or 200 µl of 0.9% NaCl as a placebo. The ribavirin dose that is fatal to 50% of mice (LD_50_) is 220 mg/(kg×d) [47]. Treatment was commenced 1 h p.i. and continued until death or day 8. Arbidol was administered once daily per os using a stomach probe. Suspensions of 15 or 30 mg/ml in 0.5% methylcellulose was prepared before each application. Animals received an arbidol dose of 75 or 150 mg/(kg×d) (100 µl suspension for a 20-g mouse) or 100 µl of 0.5% methylcellulose as a placebo. Treatment was commenced 1 day before infection and continued until death or day 8. *In vivo* toxicity of arbidol was evaluated in 26 uninfected animals treated with 0, 25, 75, 150, 300, or 600 mg/(kg×d) for 8 days. No toxic effects were observed in this dose range. T-705 was administered twice daily per os using a stomach probe. Suspensions of 0.75, 1.5, 3, or 30 mg/ml in 0.5% methylcellulose were prepared daily. Animals received a T-705 dose of 7.5, 15, 30, or 300 mg/(kg×d) (100 µl suspension twice daily for a 20-g mouse) or 100 µl of 0.5% methylcellulose twice daily. Treatment was commenced 1 h p.i. or later and continued until death or day 8.

### Titration of virus and antibodies

Infectious virus particles in blood and organ samples were determined by immunofocus assay. Organ samples were homogenized in 500 µl DMEM–2% FCS using Lysing Matrix D (MP Biomedicals) in a beat mill. Vero cells in 24-well plates were inoculated with 200 µl of serial 10-fold dilutions of sample. The inoculum was removed after 1 h and replaced by a 1%-methylcellulose–DMEM–6% FCS overlay. After 5 days of incubation, cells were fixed with 4% formaldehyde in phosphate-buffered saline (PBS), washed with water, and permeabilized with 0.5% Triton X-100 in PBS. After washing and blocking with 10% FCS in PBS, infected cell foci were detected with CCHFV nucleoprotein (NP)-specific monoclonal antibody A4 [48]. After washing, cells were incubated with peroxidase-labeled anti-mouse IgG. Foci were visualized with tetramethylbenzidine and counted.

Virus-specific antibodies in blood were detected by immunofluorescence assay (IFA) using cells infected with CCHFV strain Afg-09 2990 as an antigen. Mouse serum was inactivated for 1 h at 60°C and tested at a dilution of 1∶20.

### Clinical chemistry

Serum samples were diluted 1∶10 or higher, if required, in 0.9% NaCl and analyzed for AST and ALT activity by using commercially available colorimetric assay kits at 25°C (detection limit for undiluted serum is 2.25 U/l for AST and 2.65 U/l for ALT) (Reflotron, Roche Diagnostics). Parameters were measured for individual animals.

### Histology and immunohistochemistry

Lung, kidney, heart, spleen, brain, and liver were collected, fixed in 4% formaldehyde in PBS, and embedded in paraffin using a Leica ASP300 S tissue processor and a Leica EG1160 embedding station (Leica). Sections (4 µm) were stained with hematoxylin–eosin (H&E) or processed for immunohistochemistry (IHC). IHC sections were stained using the Ventana BenchMark XT automated staining system (Ventana Medical Systems) and Cell Conditioning solution 1 or 2 (Ventana) for 30–60 min. Sections were incubated with primary antibodies directed against B cell marker B220 (1∶400; eBioscience), apoptosis marker cleaved caspase-3 (1∶100; R&D Systems), T cell marker CD3 (1∶100; Dako), myeloid-lineage cell (e.g. macrophage) marker Iba-1 (1∶2,000; Wako Chemicals), inducible nitric oxide synthase (iNOS) expressed by activated monocyte-derived cells (1∶50; Abcam), and cell proliferation marker Ki67 (1∶250; Abcam) for 1 h. Primary antibodies were detected with anti-mouse IgG, anti-rabbit IgG, or anti-rat IgG Histofine Simple Stain MAX PO immuno-enzyme polymer (Nichirei Biosciences) and stained with 3,3′-Diaminobenzidine (DAB) substrate using the ultraView Universal DAB Detection Kit (Ventana). Cells were counterstained with hematoxylin. IHC with primary antibodies directed against CCHFV NP (monoclonal antibody A4 [48], 1∶500) and neutrophil marker Ly6G (1∶1,000; BD Bioscience) was performed manually. Sections were boiled in citrate buffer (pH 6) for 1 h and incubated with antibody at 4°C overnight. Primary antibodies were detected with anti-mouse IgG Histofine Simple Stain AP or anti-rat IgG Histofine Simple Stain MAX PO immuno-enzyme polymer and stained with Fast Red (Roche) or DAB (Sigma-Aldrich) substrate, respectively. Mayer's hematoxylin solution was used for counterstaining. Sections were coverslipped with Tissue Tek mounting medium (Sakura Finetek).

### Statistical analysis

Statistical analysis was performed with GraphPad 6.0 (GraphPad Software). Unpaired groups were compared with the two-tailed Mann–Whitney U test for continuous parameters and with two-tailed Fisher's exact test for frequencies. Survival curves were compared with the log-rank (Mantel–Cox) test.

## Results

### Optimization of IFNAR^−/−^ mouse model for antiviral testing

**Figure 1:**
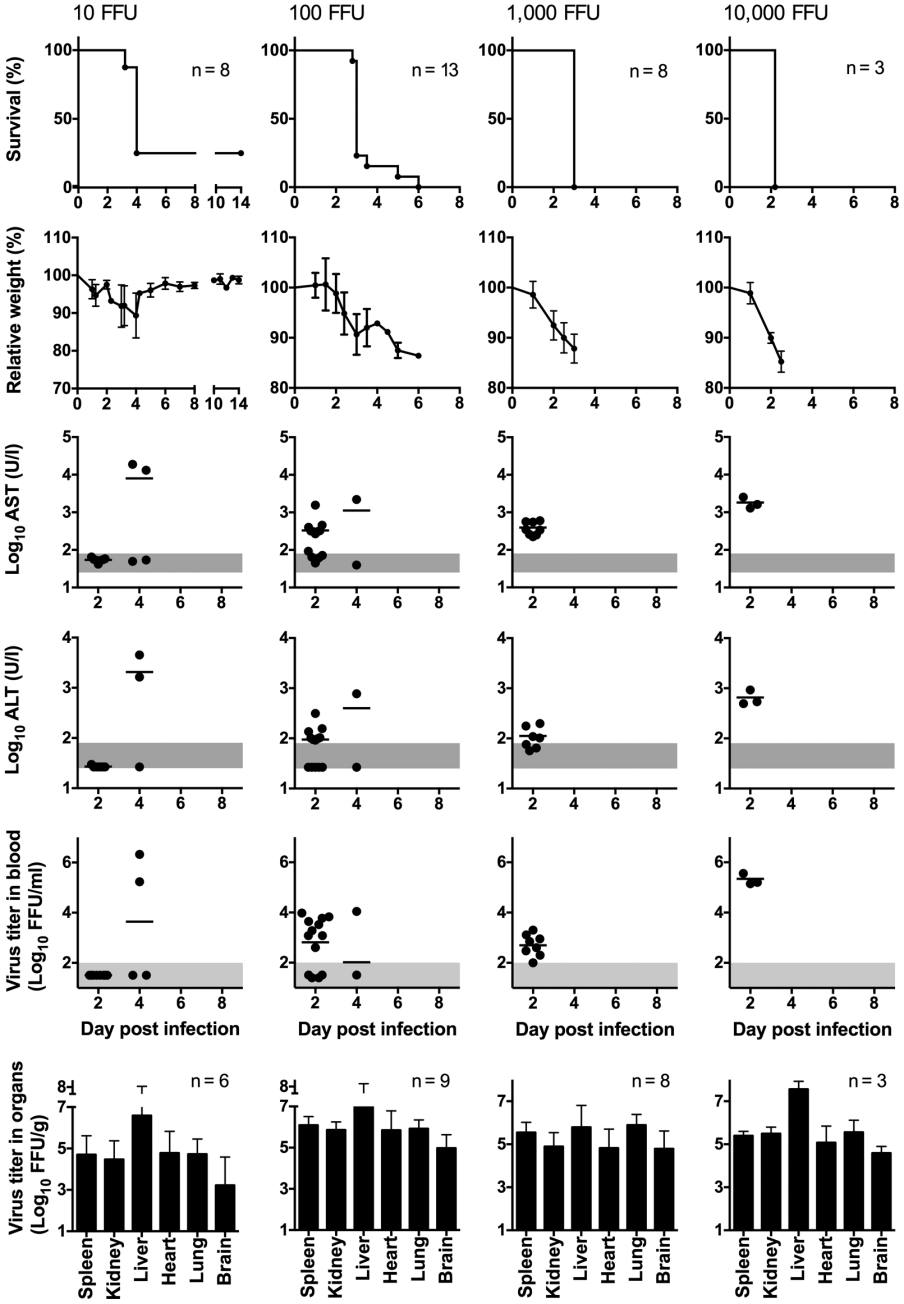
Survival, body weight, AST, ALT, and virus titer in blood and organs of IFNAR^−/−^ mice infected with different doses of CCHFV. Mice were inoculated i.p. with 10, 100, 1,000, or 10,000 FFU of virus. Organ titers were determined in animals that succumbed to the infection or had to be euthanized due to the severity of the disease. Mean and standard deviation are shown for weight and log-transformed organ titers. Vertical bars in the graphs for AST and ALT (note the log scale of the y-axis) and the log-transformed virus titers in blood represent the mean values. The data for the 100 and 1,000 FFU groups of naïve animals were pooled with corresponding data from placebo controls shown in Fig. 5 to provide more reliable estimates for the parameters. The range of viremia below the detection limit of the immunofocusassay as well as the normal reference range of AST and ALT in mice [59] are shaded in grey. Notes. 10 FFU group: The 2 surviving animals showed neither AST/ALT elevation nor viremia at day 4 (the ALT value for one animal was not determined). 100 FFU group: The animal, which died at day 6, showed no AST/ALT elevation and viremia at days 2 and 4.

Before testing antivirals in the IFNAR^−/−^ mouse model, we aimed at determining the optimal infection dose for CCHFV strain Afg09-2990 and characterizing the disease caused by this particular strain. To this end, IFNAR^−/−^ mice were infected i.p. with 0.3, 1, 3, 10, 100, 1,000, and 10,000 FFU. Animals died from the infection even after inoculation with 0.3 FFU (inoculum, died/infected: 0.3 FFU, 4/5; 1 FFU, 5/5; 3 FFU, 4/5; 10 FFU, 6/8; 100 FFU 13/13; 1,000 FFU, 8/8; 10,000 FFU, 3/3). This indicates that only a few infectious virus particles of CCHFV strain Afg09-2990 are sufficient to initiate a productive infection. A lethal outcome was consistently observed with ≥100 FFU. Therefore, the model was further characterized for the inoculation doses 10, 100, 1,000, and 10,000 FFU (Fig. 1). Animals infected with 100 or 1,000 FFU died between days 3 and 6, while animals infected with 10,000 FFU uniformly died at day 2. Before death, animals lost about 15% of body weight (Fig. 1). At day 2, the mean AST and ALT values were around 300 U/l and 100 U/l, respectively, in animals inoculated with 100–1,000 FFU. Both values were higher in the 10,000 FFU group (AST 1,600 U/l and ALT 500 U/l) (Fig. 1). AST and ALT elevations indicated cell damage, in particular of liver cells. At day 2, virus titer in blood ranged from below detection lime in the 10 FFU group, via 3 log_10_ FFU/ml in the 100 and 1,000 FFU groups, up to 5 log_10_ FFU/ml in the 10,000 FFU group (Fig. 1). At day 3, virus was found in all organs analyzed (spleen, kidney, liver, heart, lung, and brain) at titers ranging from 4–7 log_10_ FFU/g irrespective of the inoculation dose (Fig. 1). The maximum virus concentration was found in liver. As the inoculation with ≤10 FFU was not uniformly lethal and the inoculation with 10,000 FFU leaves only 2 days between infection and lethal outcome for therapeutic intervention, further experiments were conducted with a dose of 100 or 1,000 FFU.

### Histopathological correlates of disease

Lung, heart, kidney, brain, liver and spleen of CCHFV-infected IFNAR^−/−^ mice were collected at day 3 and assessed on H&E-stained sections. Virus distribution in all organs and inflammatory response in liver were visualized by IHC. Naïve IFNAR^−/−^ mice served as a control.

#### Liver

**Figure 2:**
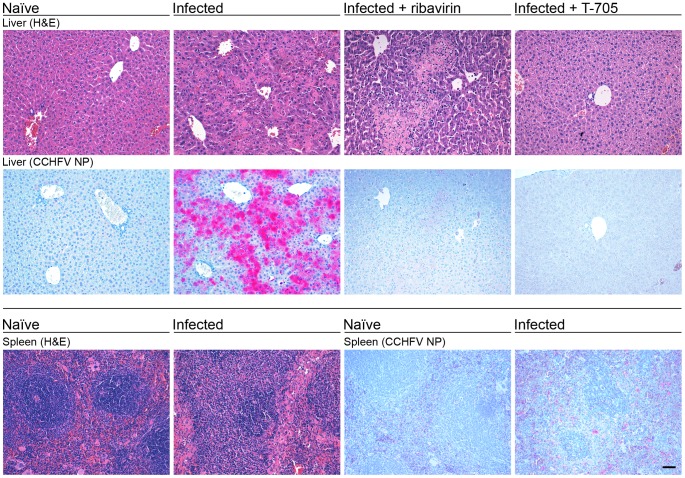
Histopathology and distribution of viral antigen in liver and spleen of naïve and CCHFV-infected IFNAR^−/−^ mice and effect of treatment with ribavirin and T-705. Animals were inoculated i.p. with 100 FFU of CCHFV. Top: Liver was collected (i) from naïve animals (ii) from animals that succumbed to the infection without treatment at day 3 p.i., (iii) that succumbed to the infection at day 9 p.i. following ribavirin treatment with 100 mg/(kg×d), and (iv) that were euthanized at day 3 p.i. during T-705 treatment with 300 mg/(kg×d). Treatment with ribavirin or T-705 was commenced 1 h p.i. and continued until death or day 8. Bottom: Spleen was collected from naïve animals and animals that succumbed to the infection without treatment at day 3 p.i. Sections were stained with H&E and the distribution of CCHFV in the tissue was visualized using a monoclonal antibody against CCHFV NP. Histopathological findings are representative for 2–3 animals that were analyzed per group. Scale bar = 50 µm.

**Figure 3:**
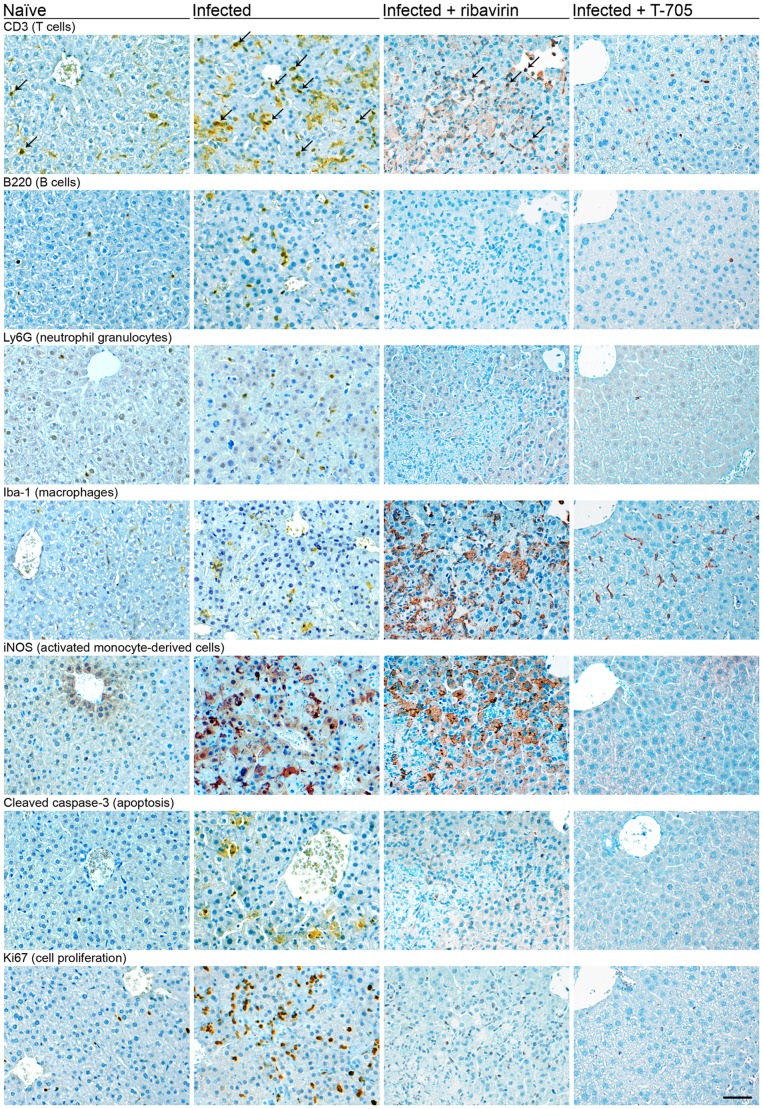
IHC for markers of inflammation, apoptosis, and proliferation in the liver of naïve and CCHFV-infected IFNAR^−/−^ mice and effect of treatment with ribavirin and T-705. Animals were inoculated i.p. with 100 FFU of CCHFV. Liver was collected (i) from naïve animals (ii) from animals that succumbed to the infection without treatment at day 3 p.i., (iii) that succumbed to the infection at day 9 p.i. following ribavirin treatment with 100 mg/(kg×d), and (iv) that were euthanized at day 3 p.i. during T-705 treatment with 300 mg/(kg×d). Treatment with ribavirin or T-705 was commenced 1 h p.i. and continued until death or day 8. Sections were stained with antibodies against CD3, B220, Ly6G, Iba-1, iNOS, cleaved caspase-3, and Ki67. T cells are marked with arrows, as the antibody against CD3 shows some spurious crossreactivity. Histopathological findings are representative for 2–3 animals that were analyzed per group. Scale bar = 50 µm.

The liver of CCHFV-infected IFNAR^−/−^ mice showed acute hepatitis with extensive bridging hepatocellular necrosis and a mild to moderate inflammatory response (Fig. 2). In the necrotic areas, accumulation of CCHFV NP in hepatocytes (Fig. 2) and cleaved caspase-3-positive (apoptotic) hepatocytes were detected (Fig. 3). Cell proliferation, as determined by staining of nuclear antigen Ki67, was increased. Although most Ki67-positive cells could be attributed to the inflammatory response, proliferation of hepatocytes was also evident (Fig. 3). The architecture of Iba-1-positive macrophages (Kupffer cells) in the necrotic areas was disturbed; the cells showed enlarged cell bodies and focal clustering (Fig. 3). In addition, iNOS-expressing activated monocyte-derived cells, which are not present in normal liver, were detectable (Fig. 3). These alterations are suggestive for monocyte/macrophage activation. The numbers of CD3-positive T cells, B220-positive B cells, and Ly6G-positive granulocytes was slightly increased (Fig. 3).

#### Spleen

The spleen of CCHFV-infected mice showed moderately decreased numbers of lymphocytes and increased amounts of cell debris in the red and white pulp. Furthermore, the number of megakaryocytes was decreased (Fig. 2). CCHFV NP was detectable in spleen cells, although the signals were much less intense than in liver (Fig. 2).

#### Lung, heart, kidney, and brain

Histopathological analysis did not reveal any obvious differences in lung, heart, kidney, and brain tissue between CCHFV-infected and naïve mice. Virus antigen level was below the detection limit of the IHC (data not shown).

In summary, IFNAR^−/−^ mice infected with CCHFV showed pathological changes in liver and spleen. Main finding was acute hepatitis with extensive necrosis, reactive proliferation of hepatocytes, mild to moderate inflammatory response, and morphological signs of monocyte/macrophage activation. CCHFV-infected and apoptotic hepatocytes clustered in the necrotic areas.

### Antiviral and toxicity testing *in vitro*

**Figure 4:**
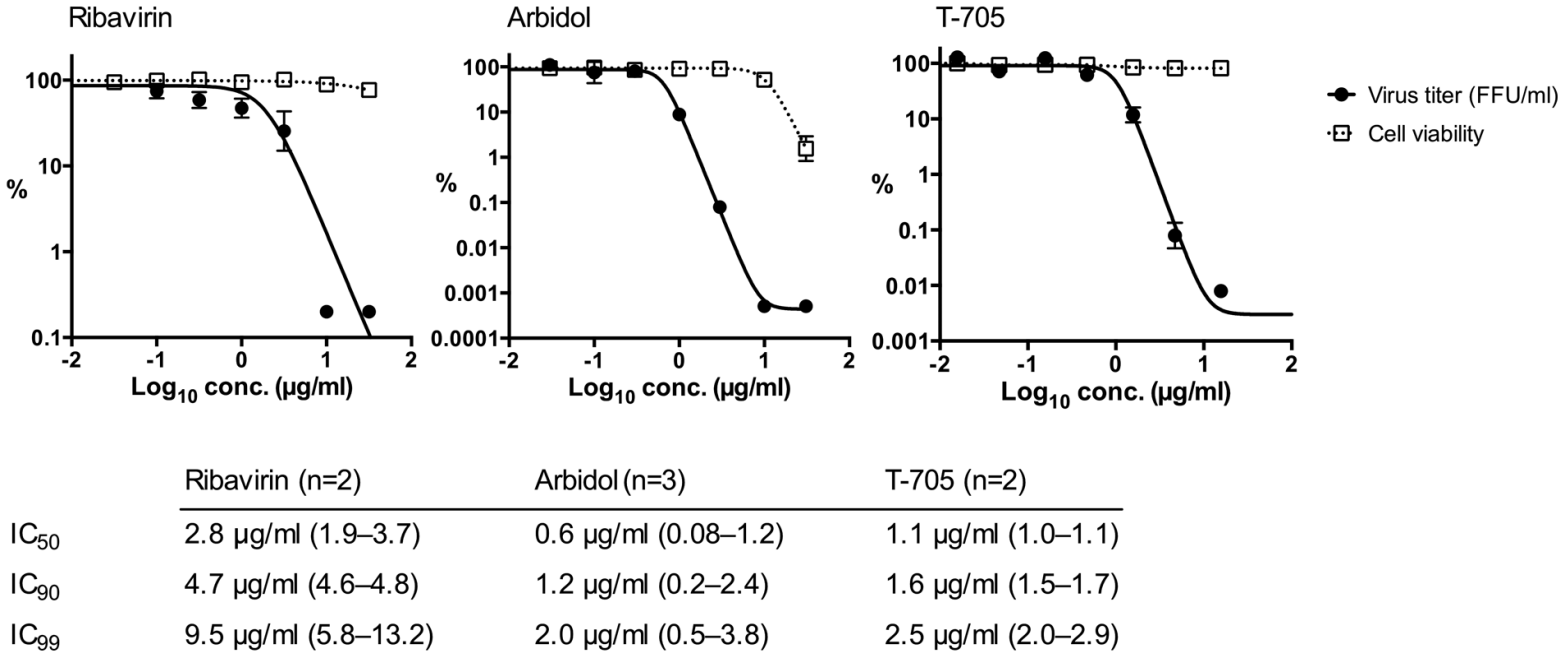
Testing of the antiviral activity of ribavirin, arbidol hydrochloride, and T-705 in cell culture. Vero E6 cells were inoculated with CCHFV at a MOI of 0.01 and compound was added 1–4 days p.i. by immunofocus assay. Cell growth and viability under compound treatment was determined by the MTT method. Dose–response curves were fitted to the data. The graphs show representative experiments with data points representing mean and standard deviation of ≥3 replicates. The IC_50_, IC_90_, and IC_99_ values were calculated from the sigmoidal functions of 2–3 independent experiments and are shown below the graphs (mean and range).

The antiviral activity of ribavirin, arbidol hydrochloride, and T-705 against CCHFV strain Afg09-2990 was tested in Vero E6 cells. All three compounds were able to suppress virus replication by 3–4 log_10_ units at concentrations of ≥10 µg/ml (Fig. 4). IC_50_ and IC_90_ values ranged from 0.6–2.8 µg/ml and 1.2–4.7 µg/ml, respectively. IC_99_ values ranged from 2.0–9.5 µg/ml. Cell toxicity in the test range as measured by MTT test was only evident for arbidol hydrochloride (ribavirin CC_50_>32 µg/ml; arbidol hydrochloride CC_50_ 8.3 µg/ml, CC_90_ 20 µg/ml; T-705 CC_50_>15 µg/ml) (Fig. 4). In conclusion, all three compounds showed a potent antiviral effect against CCHFV Afg09-2990 in cell culture. Arbidol displayed toxicity with a therapeutic index of about 10.

### Antiviral testing *in vivo*

**Figure 5:**
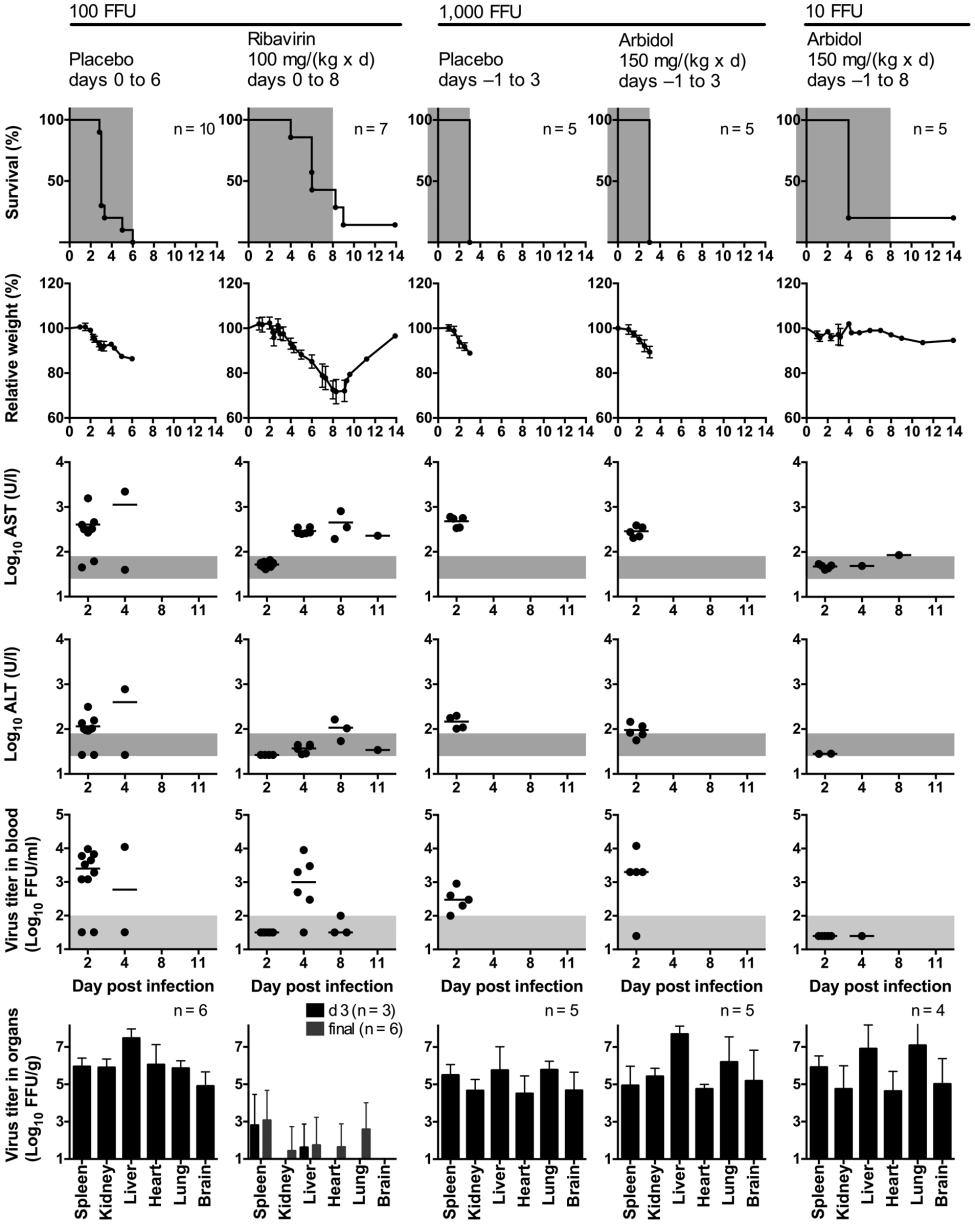
Treatment of CCHFV-infected IFNAR^−/−^ mice with ribavirin and arbidol hydrochloride. Mice were inoculated i.p. with 10, 100, or 1,000 FFU of virus. Ribavirin was administered i.p. once daily. Animals received a ribavirin dose of 100/(kg×d) or 0.9% NaCl as a placebo. Treatment was commenced 1 h p.i. and continued until death or day 8. Arbidol was administered once daily per os using a stomach probe. Animals received an arbidol dose of 150 mg/(kg×d) or 0.5% methylcellulose as a placebo. Treatment was commenced 1 day before infection and continued until death or day 8. Organ titers were determined in animals that succumbed to the infection or had to be euthanized due to the severity of the disease. In addition, three animals from the ribavirin-treated group were randomly euthanized at day 3 p.i. to determine the virus titer in organs (weight, AST, ALT, and viremia data obtained from these mice until day 3 were included in the respective graphs). Mean and standard deviation are shown for weight and log-transformed organ titers. Vertical bars in the graphs for AST and ALT (note the log scale of the y-axis) and the log-transformed virus titers in blood represent the mean values. The duration of treatment in the survival plots, the range of viremia below the detection limit of the immunofocusassay as well as the normal reference range of AST and ALT in mice [59] are shaded in grey. Notes. 100 FFU placebo group: The animal, which died at day 6, showed no AST/ALT elevation and viremia at day 4. 100 FFU ribavirin group: The surviving animal did not show detectable viremia, but AST elevation. Viremia was not determined at day 11. 10 FFU arbidol group: The surviving animal had no AST elevation and viremia at day 4. Some values for days 4, 8, and, 11 were not determined due to insufficient amount of blood.

Ribavirin was tested in comparison to a placebo group receiving the vehicle (0.9% NaCl solution) (Fig. 5). Both groups of IFNAR^−/−^ mice were infected with 100 FFU CCHFV. Although one animal survived after treatment, ribavirin did not significantly increase the survival rate (p = 0.4). However, the drug prolonged the time to death (median 3 vs. 6 days for placebo vs. ribavirin, p = 0.0007), reduced the levels of AST (p = 0.001) and ALT (p = 0.006) at day 2, reduced the virus titer in blood at day 2 (p = 0.0007), increased the weight at day 2 and 3 (p = 0.03 and p = 0.002, respectively), and reduced the terminal virus concentration in all organs when compared to placebo at day 3 (p<0.001 separately for each organ). Histopathological analysis of organs collected at day 3 from ribavirin-treated mice revealed only small disseminated foci of necrosis; most of the liver parenchyma resembled naïve mice. Markedly reduced hepatocellular necrosis correlated with low numbers of apoptotic hepatocytes (cleaved caspase-3), T-cells (CD3), B-cells (B220), and activated monocyte-derived cells (iNOS). Virus antigen-positive cells (NP) were significantly reduced in liver and spleen compared to untreated or placebo-treated mice (data not shown). However, ribavirin-treated mice that succumbed to infection on days 4–9 showed extensive bridging hepatocellular necrosis at the time of death (Fig. 2). Like in untreated mice, the necrosis was accompanied by presence of numerous Iba-1-positive macrophages (Kupffer cells), showing enlarged cell bodies and focal clustering, and iNOS-expressing activated monocyte-derived cells (Fig. 3). Both alterations are suggestive for strong monocyte/macrophage activation. However, in contrast to untreated mice, virus antigen was hardly detectable in liver tissue of the treated mice (Fig. 2), consistent with the low virus titer in all organs (Fig. 5, bottom). Thus, ribavirin reduces CCHFV load and delays disease progression, but it does not prevent terminal liver necrosis, monocyte/macrophage activation, and lethal outcome in the IFNAR^−/−^ mouse model.

Arbidol hydrochloride [75 and 150 mg/(kg×d)] was tested in comparison to a placebo group receiving the vehicle (0.5% methylcellulose) [Fig. 5 and data not shown for 75 mg/(kg×d)]. Both groups were infected with 1,000 FFU CCHFV. Mice were pretreated one day before inoculation. However, the drug changed neither survival rate and survival time, nor any of the other parameter measured. Even reducing the inoculation dose to 10 FFU had no effect when compared to the historical control group.

**Figure 6:**
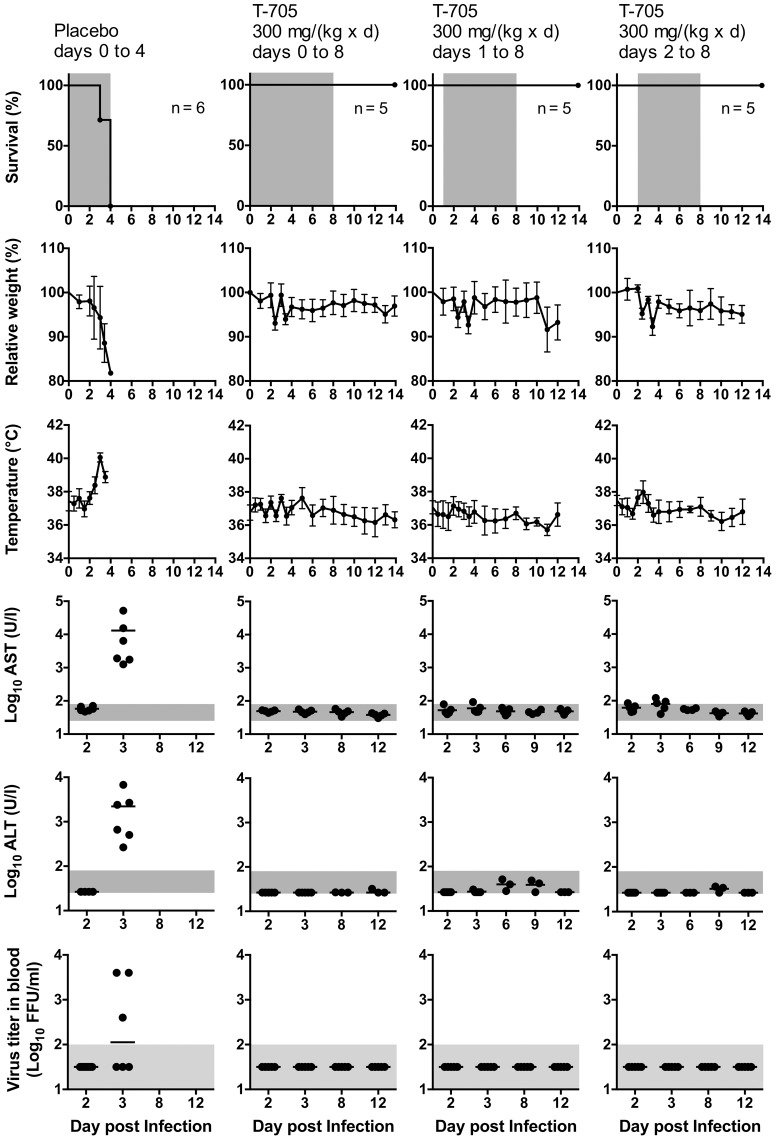
Treatment of CCHFV-infected IFNAR^−/−^ mice with T-705 and time-of-addition experiments. Mice were inoculated i.p. with 100 FFU of virus. T-705 was administered twice daily per os using a stomach probe. Animals received a T-705 dose of 300 mg/(kg×d) or 0.5% methylcellulose as a placebo. Treatment was commenced 1 h, 1 day, or 2 days p.i. and continued until death or day 8. Two animals per treatment regimen were euthanized at day 3 p.i. to determine the virus titer in organs. Mean and standard deviation are shown for weight and temperature. Vertical bars in the graphs for AST and ALT (note the log scale of the y-axis) and the log-transformed virus titers in blood represent the mean values. The duration of treatment in the survival plots, the range of viremia below the detection limit of the immunofocusassay as well as the normal reference range of AST and ALT in mice [59] are shaded in grey.

T-705 was tested in comparison to a placebo group receiving the vehicle (0.5% methylcellulose) (Fig. 6). All groups were infected with 100 FFU CCHFV. Initially, a high dose of T-705 [300 mg/(kg×d)] was tested. The drug was administered from day 0 to day 8. Placebo-treated animals died between day 3 and 4. At day 3, they showed weight loss of nearly 20%, increase in body temperature up to 40°C, AST values of 1,200–51,000 U/l, and ALT values of 260–6,700 U/l. All animals of the treatment group survived the infection and showed no signs of disease. Virus was detected neither in blood nor in the organs throughout the observation period (Fig. 6 and data not shown). Histopathology and IHC at day 3 revealed largely normal liver tissue with absence of virus antigen and inflammatory cells (Figs. 2 and 3).

To determine the efficacy of the drug at an advanced stage of the infection, time-of-addition experiments were performed (Fig. 6). Treatment with a high dose of the drug was commenced 1 day or 2 days after virus inoculation and continued until day 8. Survival was 100% in both groups and animals showed hardly any signs of disease. Only if treatment started 2 days p.i., minor changes in weight, temperature, and AST were seen at day 3. Virus remained undetectable in blood and organs in both time-of-addition groups throughout the observation period. To provide evidence for infection of the animals in the T-705 treatment groups, the development of CCHFV-specific antibodies was measured 21 days p.i. Only 1/10 (10%) of the animals treated post-exposure, but 10/10 (100%) of the animals treated from day 1 or 2 p.i. developed antibodies, indicating that virus replication under post-exposure treatment with T-705 was even not sufficient to elicit antibodies.

**Figure 7:**
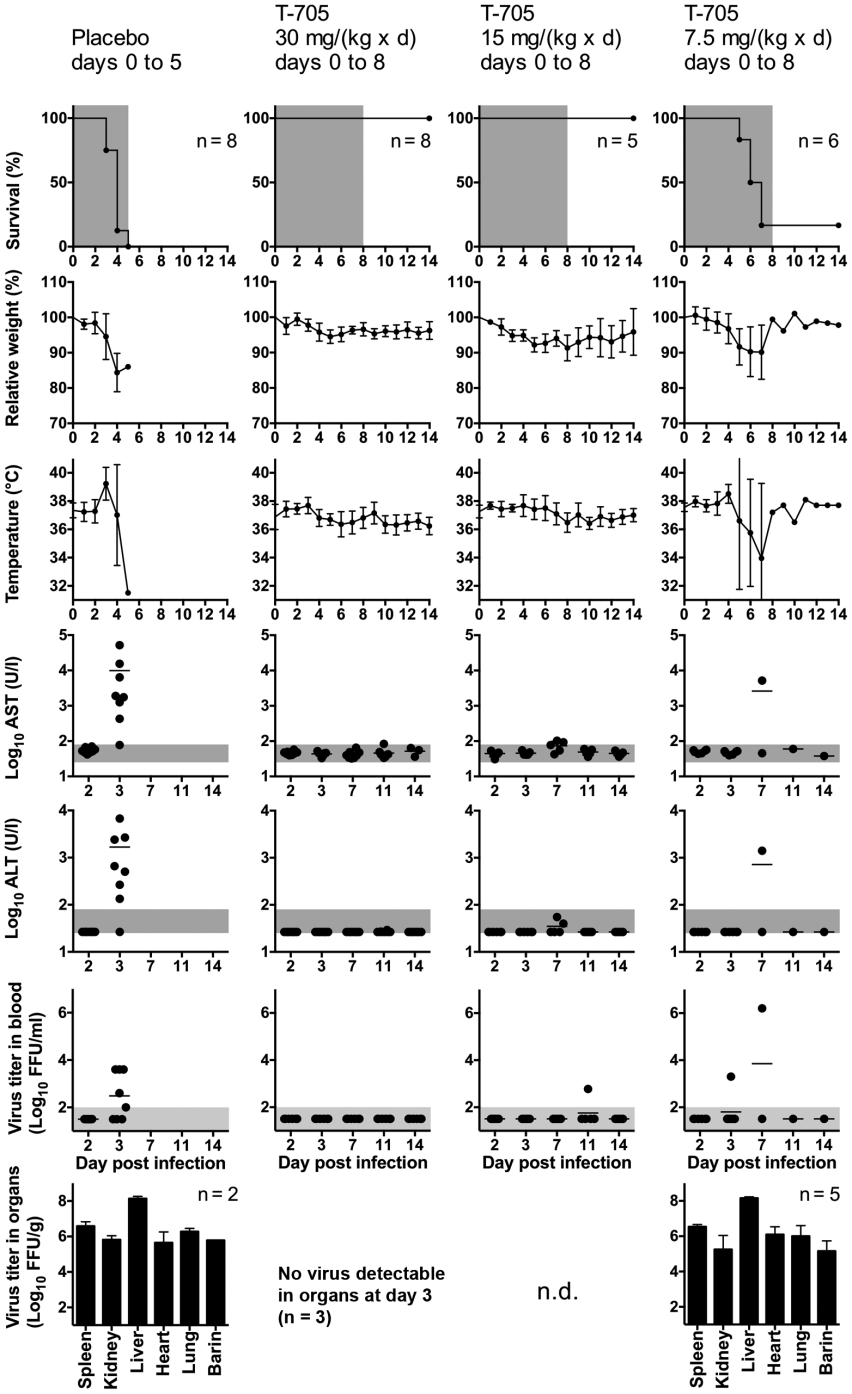
Definition of the lowest effective dose of T-705. Mice were inoculated i.p. with 100 FFU of virus. T-705 was administered twice daily per os using a stomach probe. Animals received a T-705 dose of 30, 15, or 7.5 mg/(kg×d) or 0.5% methylcellulose as a placebo. Treatment was commenced 1 h p.i. and continued until death or day 8. The virus titer in organs was determined for animals that succumbed to the infection and for 3 animals of the 30 mg/(kg×d) group that were euthanized at day 3. Organ analysis was not done for the 15 mg/(kg×d) group (n.d.). Mean and standard deviation (or range for n = 2) are shown for weight, temperature and log-transformed organ titers. Vertical bars in the graphs for AST and ALT (note the log scale of the y-axis) and the log-transformed virus titers in blood represent the mean values. The duration of treatment in the survival plots, the range of viremia below the detection limit of the immunofocusassay as well as the normal reference range of AST and ALT in mice [59] are shaded in grey. Notes. Placebo group: The animal, which died at day 5, showed no AST/ALT elevation and viremia at day 3. 7.5 mg/(kg×d) T-705 group: The surviving animal showed neither AST/ALT elevation nor viremia at any time.

To define the lowest effective dose of T-705, animals received 30, 15, or 7.5 mg/(kg×d) T-705 or 0.5% methylcellulose as a placebo (Fig. 7). Treatment was commenced 1 h p.i. and continued until death or day 8. All animals of the 30 and 15 mg/(kg×d) treatment groups survived and showed hardly any signs of disease. Virus was detected at low level only in blood of one animal of the 15 mg/(kg×d) treatment group at day 11 (Fig. 7). A dose of 7.5 mg/(kg×d) did not prevent a lethal outcome, although it prolonged the time to death (p = 0.0007), and reduced the levels of AST (p = 0.0007) and ALT (p = 0.004) at day 3.Taken together, T-705 is highly efficient against CCHFV in the IFNAR^−/−^ mouse model.

### Combination of ribavirin and T-705 *in vitro* and *in vivo*

**Figure 8:**
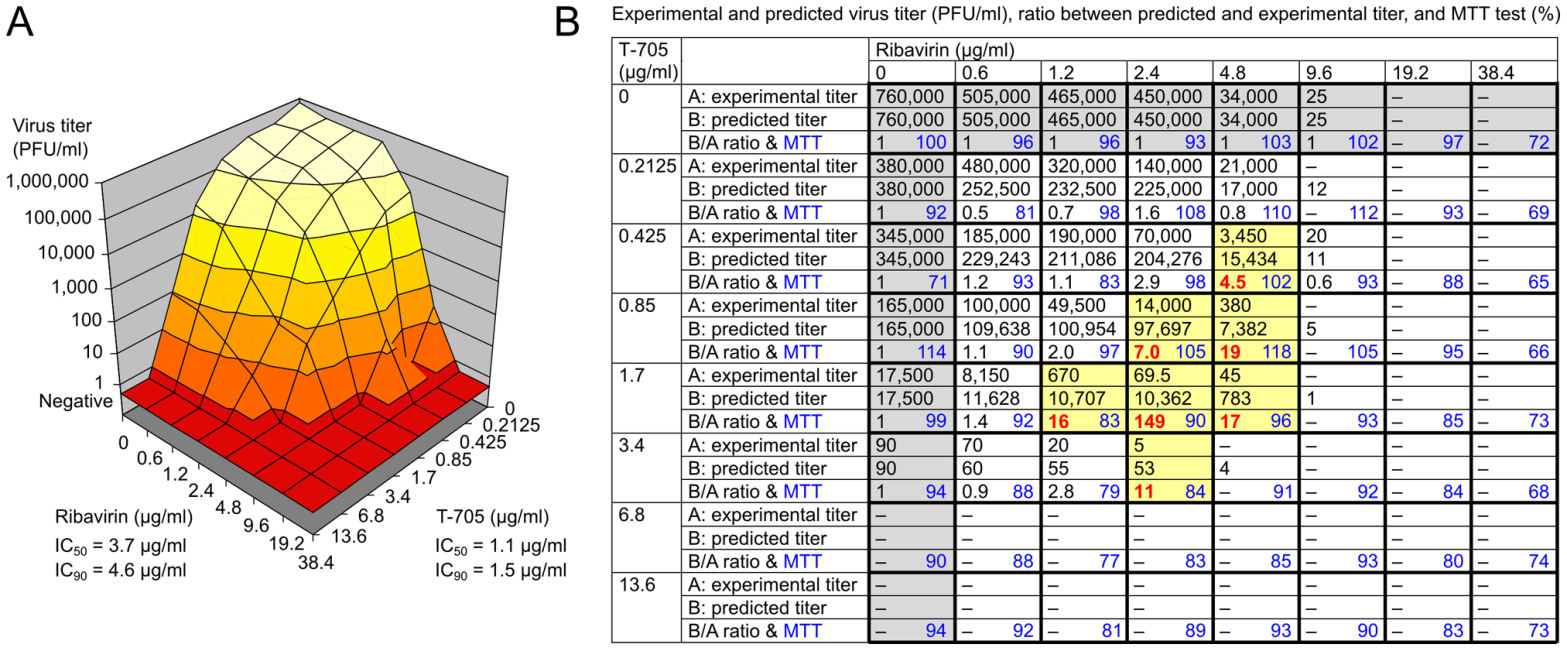
Testing of the antiviral activity of combination of ribavirin and T-705 in cell culture. Vero E6 cells were inoculated with CCHFV at a MOI of 0.01 and the compounds were added 1×8 drug combination matrix was designed as described in Materials and Methods. Concentration in cell culture supernatant of infectious virus particles was measured 3 days p.i. by immunofocus assay. Cell growth and viability under compound treatment was determined by the MTT method. (A) Dose–response surface for combination of ribavirin and T-705. Sigmoidal dose–response curves were fitted to the single-drug data and the IC_50_, and IC_90_ values for ribavirin and T-705 were calculated from the functions. (B) In analogy to the MacSynergy II program [44], [45], a three-dimensional approach was used to identify areas where observed effects are greater (synergy) or less (antagonism) than predicted by the Bliss independence model. To this end, the ratio between predicted virus titer and observed virus titer was calculated for each drug combination. A ratio >1 indicates synergy (i.e. the virus titer predicted for additive effect is higher than the experimentally determined virus titer), a ratio <1 indicates antagonism (i.e. for the virus titer for predicted additive effect is lower than the experimentally determined virus titer). Drug combinations showing a clear difference between experimental and predicted titer (≥4-fold) are marked in yellow with the ratio indicated in red. MTT values are shown in blue for each drug combination.

Ribavirin is currently in clinical use for treatment of CCHF [16]–[19]. Therefore, it is important to know if T-705 could be given in combination with ribavirin and how both drugs interact. First, the antiviral activity of 64 combinations of ribavirin and T-705 was determined in cell culture. The 8×8 concentration matrix was designed around the IC_90_ values of both drugs as determined above. Infectious virus particles were measured 3 days p.i. by immunofocus assay and cell viability was determined by the MTT method. The dose–response surface demonstrates that combinations of ribavirin and T-705 exhibit strong antiviral effects with suppression of virus replication by >5 log units (Fig. 8A). Possible antagonistic or synergistic effects were evaluated using the Bliss independence model in analogy to the algorithms of the MacSynergy II program [44], [45]. This analysis revealed clear synergistic effects when the drugs were combined in concentrations around their IC_90_. In this area of the matrix, the experimental virus titer was up to 2 log units lower than the titer predicted according to the Bliss independence model for additive effect (Fig. 8B). The MTT test did not reveal drug toxicity over the whole matrix (Fig. 8B).

**Figure 9:**
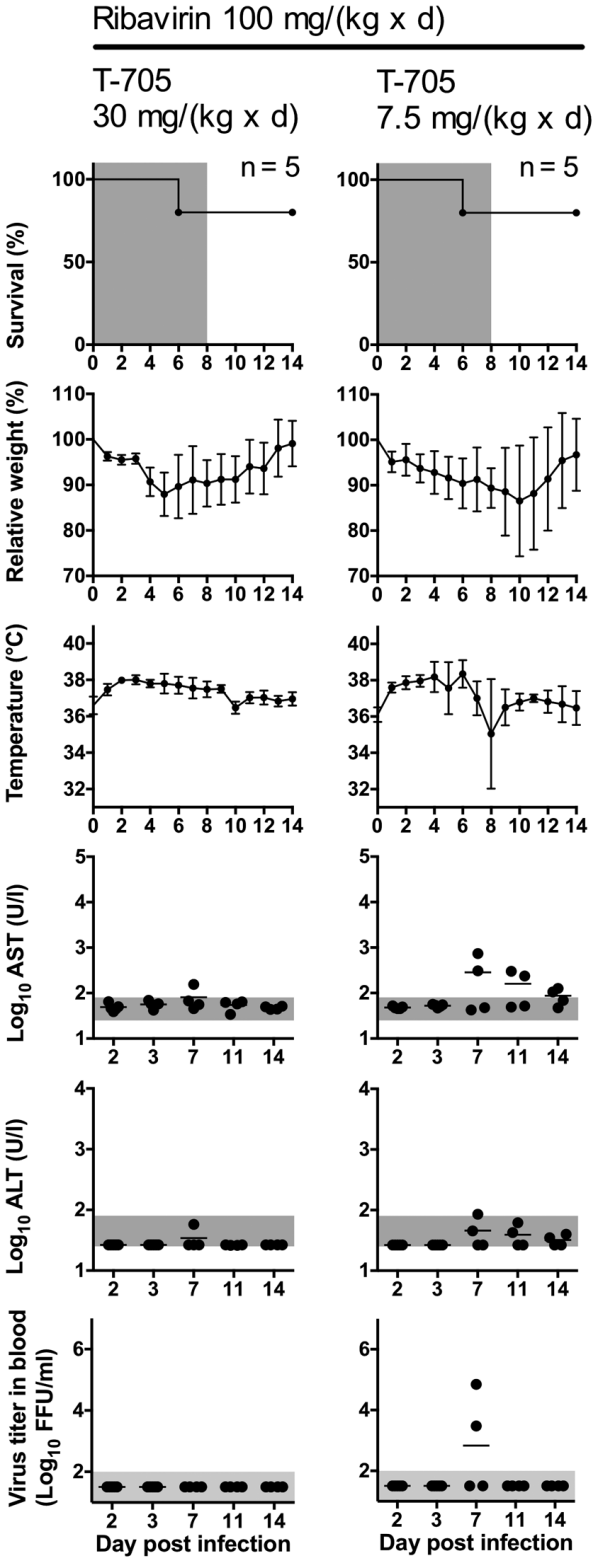
Treatment of mice with a combination of ribavirin and T-705. Mice were inoculated i.p. with 100 FFU of virus. T-705 was administered twice daily per os using a stomach probe. Ribavirin was administered i.p. once daily. Animals received a T-705 dose of 30 or 7.5 mg/(kg×d) in combination with a ribavirin dose of 100 mg/(kg×d). The placebo controls are shown in Fig. 7, as both experiments have been conducted in parallel. Treatment was commenced 1 h p.i. and continued until death or day 8. Mean and standard deviation are shown for weight and temperature. Vertical bars in the graphs for AST and ALT (note the log scale of the y-axis) and the log-transformed virus titers in blood represent the mean values. The duration of treatment in the survival plots, the range of viremia below the detection limit of the immunofocusassay as well as the normal reference range of AST and ALT in mice [59] are shaded in grey. Notes. The two animals that died at day 6 had no detectable virus in organs or blood and no AST/ALT elevation.

To test the effects of drug combination *in vivo*, animals received a T-705 dose of 30 or 7.5 mg/(kg×d) in combination with a ribavirin dose of 100 mg/(kg×d). The 30 mg/(kg×d) T-705 dose, which is protective upon single-drug administration, was chosen to test if addition of ribavirin interferes with T-705 efficacy. To explore if combination of two sub-effective doses may result in an effective treatment, 7.5 mg/(kg×d) T-705 and 100 mg/(kg×d) ribavirin were co-administered. None of the parameters in the 30 mg/(kg×d) T-705 plus 100 mg/(kg×d) ribavirin group (Fig. 9, left) was statistically significantly different from the parameters of the 30 mg/(kg×d) T-705 single-drug group (Fig. 7). On the other hand, the combination of a 7.5 mg/(kg×d) dose of T-705 with a 100 mg/(kg×d) dose of ribavirin (Fig. 9 right) improved the survival rate compared to single-drug treatments (Figs. 5 and 7), although the increase did not reach statistical significance (p = 0.08 for T-705+ribavirin vs. T-705 alone, and p = 0.07 for T-705+ribavirin vs. ribavirin alone; two-tailed Fisher's exact test). In conclusion, T-705 and ribavirin exert synergistic effects according to the Bliss independence model when combined in concentrations around their IC_90_ *in vitro*. Co-administration of both drugs in the animal model suggests that a combined treatment yields beneficial rather than adverse effects.

## Discussion

In this study, we have used IFNAR^−/−^ mice as an *in vivo* model to evaluate the efficacy of antivirals against CCHFV. Main pathological alteration in mice infected with the recently isolated CCHFV strain Afg09-2990 was acute hepatitis with extensive necrosis, reactive proliferation of hepatocytes, mild to moderate inflammatory response, and morphological signs of monocyte/macrophage activation. CCHFV-infected and apoptotic hepatocytes were found in the necrotic areas. Ribavirin, arbidol hydrochloride, and T-705 were active against CCHFV Afg09-2990 in cell culture. However, arbidol hydrochloride was inactive *in vivo*. Ribavirin was partially active, while T-705 was highly efficient in the mouse model. The latter drug was effective even if the window for therapeutic intervention was less than 2 days.

Three mouse models for CCHF have been described in the past: the neonatal mouse model, STAT1^−/−^ mice, and IFNAR^−/−^ mice [9]–[12]. The latter models take advantage of the defect in the innate immune response, which apparently is essential to protect mice from productive CCHFV infection. In all three models, mice are dying from the infection within a few days. We prefer to work with IFNAR^−/−^ mice, as the genetic defect concerns only the interferon type I signaling, while STAT1 deficiency prevents the upregulation of genes due to a signal by either type I or type II interferons and neonatal mice are immunologically tolerant (neonatal tolerance). IFNAR^−/−^ mice are highly susceptible to CCHFV infection. The inoculum sufficient to initiate a productive infection is very low — 0.3 FFU — which presumably corresponds to just a few infectious virus particles. This is consistent with experiments in AG129 mice lacking interferon type I and type II receptors, in which an inoculum of 0.1 FFU of lymphocytic choriomeningitis virus was sufficient to infect the animals [49].

It has been shown very recently that the IFNAR^−/−^ model mimics hallmarks of human CCHF disease [12]. These findings are extended here by a more detailed immunhistopathological analysis of the liver. Histopathology, virus load measurement, antigen staining in various organs, and the measurement of AST and ALT demonstrate that the liver is the major target organ of CCHFV. Although virus was found in all organs, the titer in liver is the highest and exceeds 7 log_10_ FFU/g in some experiments. The higher virus titer in liver compared to other organs may explain why the IHC analysis for virus antigen revealed clearly positive cells only in the liver, while the signals in other organs were weak or absent. In exceptional cases, AST and ALT values reached 10,000 U/l and 1,000 U/l, respectively, demonstrating massive liver cell damage. However, while ALT is specific for this organ, AST is also present at high level in the heart, skeletal muscle, kidneys, brain, and red blood cells [50]. Therefore, the high AST/ALT ratio may also indicate extrahepatic cell damage. Overall, the histological and biochemical findings are compatible with the diagnosis of a fulminant liver damage. Our findings are also in agreement with the pathological observations in human CCHF; hepatocellular necrosis with hyperplastic and hypertrophic Kupffer cells and mild or absent inflammatory cell infiltrates is the prominent histopathological finding in humans [3]. Two new aspects, which may provide some clues as to the pathophysiology of CCHF, are noteworthy. First, in the necrotic lesions of the liver, both CCHFV-infected hepatocytes and apoptotic hepatocytes clustered. This may suggest that CCHFV-infected cells undergo apoptosis and necrosis. As the inflammatory response was only mild to moderate, a direct cytopathic effect of the virus on hepatocytes may be involved in the induction of apoptosis and necrosis. Importantly, this hypothesis has been raised in early IHC studies on humans with CCHF as well [3]. Secondly, activated Iba-1-positive macrophages and activated monocyte-derived cells expressing iNOS were found in the liver. These cells may play a crucial role in the strong proinflammatory immune responses following CCHFV infection, as demonstrated by significant increases of serum proinflammatory cytokines and chemoattractant molecules in IFNAR^−/−^ and STAT1^−/−^ mice, as well as in humans [6]–[8], [10], [12].

In this study, we have employed the IFNAR^−/−^ mouse model for testing antivirals against CCHFV *in vivo*. Ribavirin is the standard treatment in human CCHF — although its clinical efficacy is not proven [16]–[19] — and shows beneficial effects in the neonatal and STAT1^−/−^ mouse models [9], [10]. Therefore, we first evaluated the IFNAR^−/−^ mouse model using this drug. The survival time was prolonged, while the survival rate was not increased, which largely corresponds to the results of the high-dose challenge experiments in STAT1^−/−^ mice [10]. Despite the delay in disease progression and the reduction in virus load in blood and organs as evidenced by virus titration and IHC, ribavirin was not able to prevent the lethal pathophysiological cascade. Importantly, the development of terminal liver necrosis with marked monocyte/macrophage activation in the virtual absence of virus in the organ demonstrates that host pathways, once they are triggered by the virus, mediate pathology and death irrespective of the presence of the trigger.

The second compound tested was arbidol hydrochloride, a broad-spectrum antiviral drug in clinical use against flu [20]–[24]. Arbidol hydrochloride efficiently suppressed CCHFV in cell culture. However, no beneficial effects in the IFNAR^−/−^ mouse model were observed. The drug was administered via the same route but at higher dose than in previous studies that showed beneficial effects against influenza A, coxsackie B, and hantaan virus in mice [20], [22]. The compound had significant toxicity at higher concentrations in cell culture. It is conceivable that its antiviral effect *in vitro* is at least partially attributable to general cell toxic effects that are not detected in the MTT assay used to assess cell viability. Therefore, it might be that the *in vitro* data overestimate the true antiviral effect of the drug against CCHFV. In addition, arbidol is extremely hydrophobic, which may reduce its oral bioavailability in mice. It might be worth testing arbidol in pharmaceutical formulations with enhanced solubility in future [51].

An important observation in our study is the strong antiviral effect of T-705 against CCHFV in cell culture and in the IFNAR^−/−^ mouse model. This compound has been shown to be highly active against a range of viruses *in vitro* and *in vivo*, including orthomyxoviruses, arenaviruses, and bunyaviruses of the genera *hantavirus* and *phlebovirus* [28]–[34]. Therefore, its activity against CCHFV, a bunyavirus of the genus *nairovirus*, is not unexpected. However, in view of the low or lacking potency of ribavirin and arbidol *in vivo* — both of which have almost the same IC_50_ and IC_90_ values than T-705 — the high *in vivo* potency of T-705 is surprising. The IC_50_ for CCHFV is 5–30 times lower than the IC_50_ values for other bunyaviruses [35], which may indicate that this virus is particularly sensitive to T-705. Even if it was given 2 days before the expected time of death, the animals survived and hardly showed signs of disease. If given immediately post-exposure, the drug suppresses virus replication below the level required to elicit antibodies. We could reduce the dose by a factor of 20 [from 300 to 15 mg/(kg×d)] with the drug still showing post-exposure efficacy.

The mode of action of T-705 against CCHFV is not known. In analogy to other segmented negative strand viruses, T-705-ribofuranosyl-5′-triphosphate may be incorporated into the nascent RNA strand and inhibit further strand extension or induce lethal mutagenesis [36]–[40]. How ribavirin acts against CCHFV is still not known, although the drug is in clinical use since decades. Several mechanisms have been proposed for other viruses: it may be incorporated into the virus RNA causing lethal mutagenesis [52], interfere with capping [53], inhibit the viral RNA polymerase [54], [55] or inhibit the host cell enzyme inosine monophosphate dehydrogenase (IMPDH) resulting in reduced GTP levels [56]–[58]. T-705-ribofuranosyl-5′-monophosphate was 150 times weaker than ribavirin-5′-monophosphate in its IMPDH inhibitory effect, suggesting that IMPDH is not a major target enzyme for T-705 [39], [40]. Given that the mode of action of both drugs is poorly understood, it is difficult to predict how they interact. However, as ribavirin is the standard drug for treatment of CCHF [16]–[19], a ribavirin/T-705 combination treatment would be an obvious option in clinical practice. Our experiments suggest that both drugs do not act in an antagonistic manner *in vitro* and *in vivo*. According to the Bliss independence model there is even evidence for synergistic interaction *in vitro* and the experiments in the animal model point to a beneficial rather than adverse interaction *in vivo*. In conclusion, our data hold promise for clinical efficacy of T-705 or ribavirin/T-705 combination treatment in human CCHF.
